# Position matters: light repatterns m^6^A to tune anthocyanin accumulation in roses

**DOI:** 10.1093/plphys/kiag125

**Published:** 2026-03-02

**Authors:** Benjamin J M Tremblay

**Affiliations:** Assistant Features Editor, Plant Physiology, American Society of Plant Biologists; The Sainsbury Laboratory, University of East Anglia, Norwich Research Park, Norwich NR4 7UH, United Kingdom

Roses have played a significant part in human history, appearing in some of the earliest written records. Beyond their aesthetic appeal, they have carried symbolic meaning across millennia, including in the religions and cultures of many societies (Goody 1993). Various traits have been bred over time, leading to the tens of thousands of rose varieties cultivated today. One such trait, petal coloration, reflects the accumulation (or absence) of pigments such as anthocyanins and carotenoids. Pigment production is tightly regulated during flower development and in response to environmental cues, including light (Winkel-Shirley 2001). Understanding how pigment biosynthesis is controlled is therefore of major economic importance for rose breeders, both to enable the creation of new varieties and to predict how environmental conditions influence coloration. For example, some cultivars produce inadequate pigmentation when light is limiting.

Anthocyanin biosynthesis, a major contributor to rose petal coloration, proceeds through a multistep pathway involving several enzymes acting in sequence. In hybrid tea rose (*Rosa hybrida*), the expression of anthocyanin biosynthesis genes in petals is regulated spatially and temporally by transcription factors that form MYB-bHLH-WD40 (MBW) complexes (Lloyd et al. 2017). In some cultivars, such as the light-sensitive *R. hybrida* cv. Spectra, light helps prevent rapid turnover of the central photomorphogenesis protein RhHY5, which in turn promotes binding of the MBW component RhMYB114a to the promoters of anthocyanin biosynthesis genes, increasing their expression (Yan et al. 2023). By contrast, light-insensitive cultivars such as *R. hybrida* cv. Samantha do not require light to produce anthocyanins.

Despite a working model of the transcription factor cascade that regulates anthocyanin biosynthetic gene transcription in *R. hybrida* (Yan et al. 2023), this network only partially explains pigment accumulation. Additional factors are likely involved, including post-transcriptional layers of regulation. Insights from other species provide clues: during strawberry fruit ripening, which involves anthocyanin production, transcript stability of anthocyanin biosynthesis genes has been suggested to be regulated by the RNA modification N^6^-methyladenosine (m^6^A) (Zhou et al. 2021). Such observation raises the question of whether similar RNA modifications also contribute to rose coloration.

Recently in *Plant Physiology*, Gao et al. (2026) used m^6^A-seq to profile the m^6^A epitranscriptome of *R. hybrida* cv. Spectra petals under light and dark conditions and found that changes in m^6^A distribution were enriched among transcripts of anthocyanin biosynthesis genes. Among these genes, the anthocyanin synthase gene *RhANS* showed higher transcript levels and m^6^A abundance within the coding region under light. By contrast, in the dark (where *RhANS* transcript levels are lower) m^6^A was more abundant within the 3ʹ UTR. Differences in transcript abundance between light and dark were due in part to the increased transcript stability under light, which was accompanied by increased translation efficiency. Together, these findings suggest a link between m^6^A patterning and anthocyanin biosynthesis.

To investigate the possible connection between m^6^A modification and anthocyanin biosynthesis, the authors first transiently overexpressed *RhANS* in *R. hybrida* cv. Spectra petal discs under light, which resulted in increased pigmentation. This result confirmed that increased *RhANS* expression is a key contributor to light-induced pigmentation. Next, the authors identified 2 candidate m^6^A demethylases, *RhALKBH10A* and *RhALKBH10B*, both of which were transiently downregulated in petals after initial exposure to light. Using virus-induced gene silencing (VIGS), the authors showed that suppressing the expression of the m^6^A demethylases led to an increase in *RhANS* transcript levels and m^6^A abundance in the coding region, resulting in pigmentation of petal discs. This increase in pigmentation was abrogated when *RhANS* was additionally silenced in the VIGS experiments. In summary, the data support a model in which light represses *RhALKBH10A/B*, allowing accumulation of m^6^A in the coding region. In addition, m^6^A is reduced in the 3ʹ UTR by some yet-unknown mechanism, and together this repatterning of m^6^A leads to increased *RhANS* stability and translation and boosting anthocyanin accumulation (Fig. 1).

**Figure 1:**
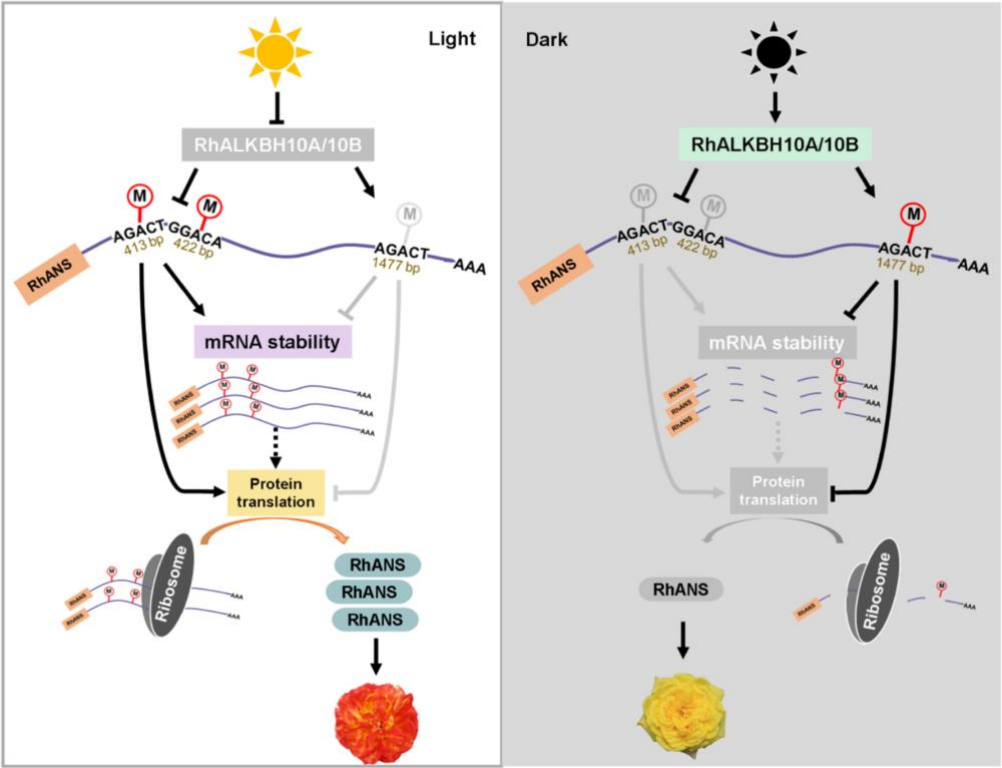
Proposed model for the role of m^6^A in light-induced anthocyanin biosynthesis in *R. hybrida* cv. Spectra. In the dark, the m^6^A demethylases RhALKBH10A and RhALKBH10B prevent m^6^A accumulation in the coding region of the anthocyanin synthase *RhANS* transcript and instead promote m^6^A accumulation in the 3ʹ UTR, reducing transcript stability. After exposure to light, expression of these demethylases is suppressed, triggering a shift in m6A patterning that increases transcript stability and translation efficiency, ultimately promoting petal pigmentation. Adapted from Fig. 6 of Gao et al. (2026).

The findings of Gao et al. further expand our understanding of the regulation of light-induced pigmentation in *R. hybrida* cv. Spectra by revealing a post-transcriptional regulatory layer that tunes transcript abundance of anthocyanin biosynthesis genes. While this work adds to our understanding of rose pigmentation, it also leaves open questions. For example, although the authors demonstrated the importance of *RhANS* for pigmentation in the light-sensitive cultivar, they observed low *RhANS* expression in the light-insensitive *R. hybrida* cv. Samantha cultivar, suggesting that cultivar-specific regulatory mechanisms may control anthocyanin accumulation and that *RhANS* transcript abundance may not be a key determinant of pigmentation across all roses.

Finally, this study has broad implications for m^6^A function in plants. Although m^6^A has been shown to play an important role in mediating transcript stability during development and stress responses, the impact of m^6^A patterning is less well understood. Gao et al. found that m^6^A within the coding region of the *RhANS* transcript was associated with increased transcript stability and translation efficiency, whereas m^6^A within the 3ʹ UTR was associated with decreased transcript stability. It remains to be seen whether these associations are shared by other transcripts, including in other species, and what mechanisms allow m^6^A position to differentially influence these processes.

Recent related articles in *Plant Physiology*:

- Guan et al. (2025) reviewed the current knowledge of the regulation of rose petal size.
- Gao et al. (2025) found that transcript stability and translation efficiency of volatile biosynthesis genes in tomato were dynamically regulated by m^6^A, contributing to fruit flavor during ripening.

## Data Availability

None required.
